# Redesign of a computerized clinical reminder for colorectal cancer screening: a human-computer interaction evaluation

**DOI:** 10.1186/1472-6947-11-74

**Published:** 2011-11-29

**Authors:** Jason J Saleem, David A Haggstrom, Laura G Militello, Mindy Flanagan, Chris L Kiess, Nicole Arbuckle, Bradley N Doebbeling

**Affiliations:** 1 VA HSR&D Center of Excellence on Implementing Evidenced-Based Practice (CIEBP), Richard L. Roudebush VA Medical Center (11-H), 1481 West Tenth St, Indianapolis, IN, 46202, USA; 2 Center for Health Services Research, Regenstrief Institute, Inc., 410 West Tenth St, Suite 2000, Indianapolis, IN, 46202, USA; 3 Center for Health Services & Outcomes Research, Indiana University (IU), 410 West Tenth St, Suite 2000, Indianapolis, IN, 46202, USA; 4 Department of Electrical & Computer Engineering, School of Engineering and Technology, 723 West Michigan Street, SL 160, Indiana University-Purdue University at Indianapolis (IUPUI), Indianapolis, IN, 46202, USA; 5 Department of Medicine, Indiana University (IU) School of Medicine, 545 Barnhill Drive, EH 317, Indianapolis, IN, 46202, USA; 6 Applied Decision Science, 1776 Mentor Ave., Suite 424, MB#118, Cincinnati, OH, 45212, USA; 7 University of Dayton Research Institute, University of Dayton, 300 College Park, Dayton, 45469, OH, USA

## Abstract

**Background:**

Based on barriers to the use of computerized clinical decision support (CDS) learned in an earlier field study, we prototyped design enhancements to the Veterans Health Administration's (VHA's) colorectal cancer (CRC) screening clinical reminder to compare against the VHA's current CRC reminder.

**Methods:**

In a controlled simulation experiment, 12 primary care providers (PCPs) used prototypes of the current and redesigned CRC screening reminder in a within-subject comparison. Quantitative measurements were based on a usability survey, workload assessment instrument, and workflow integration survey. We also collected qualitative data on both designs.

**Results:**

Design enhancements to the VHA's existing CRC screening clinical reminder positively impacted aspects of usability and workflow integration but not workload. The qualitative analysis revealed broad support across participants for the design enhancements with specific suggestions for improving the reminder further.

**Conclusions:**

This study demonstrates the value of a human-computer interaction evaluation in informing the redesign of information tools to foster uptake, integration into workflow, and use in clinical practice.

## Background

The role of human factors and human-computer interaction (HCI) methods in the development of health information technology (IT), including electronic health records (EHRs) and computerized clinical decision support (CDS), is underutilized [1-3]. Incorporating these methods can improve usability of these tools, including reduced workload and increased user satisfaction [3-7]. Methods for HCI, which is a domain of the field of human factors and ergonomics, include both field-based and laboratory-based approaches. Each approach has inherent advantages and disadvantages. Field methods, such as rapid ethnography and interviews, can capture the complexity and preserve the context of the work environment within which health IT is implemented. Laboratory-based methods, on the other hand, allow for the manipulation of experimental conditions and measurement of dependent variables in a carefully controlled setting (i.e., without introducing extraneous variability). Thus, an ideal design is a multi-method approach that leverages the advantages of both field and laboratory methods. This paper reports on the laboratory portion of such a multi-method approach to the incorporation of human factors methods in the study of CDS.

Based on an extensive field study conducted across multiple sites to understand common barriers to the use of CDS for colorectal cancer (CRC) screening [8,9], we tested a redesigned CRC screening clinical reminder in a controlled laboratory simulation experiment. Our previous field study resulted in nine themes that relate to integrating CDS into workflow. Themes included the following: 1) coordination of outside results; 2) coordination between primary and specialty care; 3) data organization and presentation; 4) just-in-time provider and patient education; 5) interface flexibility; 6) technological enhancements; 7) role assignments; 8) organizational issues; 9) and connecting decision support to quality reporting. Each of the nine themes that emerged corresponds to barriers to integrating CDS into workflow. Based on these barriers, three design features were selected as the most promising. They were judged to be likely to demonstrate high pay-off in terms of improvements to both design of CDS prototypes and their likely integration into workflow, and to be feasible in the short-term. These needed design features include the following: (1) integrating outside results; (2) improving data organization and presentation and (3) providing just-in time education for patients and cognitive support to providers when and where needed. We prototyped a redesign of the Veterans Health Administration's (VHA's) computerized clinical reminder for CRC screening that included a timeline visual to address (1) and (2), as well as a patient education resource to address (3).

The computerized clinical reminders are the main form of CDS in the VHA's EHR, known as the Computerized Patient Record System (CPRS). We chose to focus this work on CRC screening because there is a robust evidence-base to support the efficacy of CRC screening [10]; yet, rates of CRC screening are sub-optimal [11]. In our previous field study [8,9], we observed providers searching through numerous screens in the EHR to obtain the information they needed, which in many cases was characterized as frustrating and time-consuming by providers. Providers sometimes missed important information (e.g., previous CRC screening results) or relied on patient memory, which could be inaccurate. Our goal was to reduce cognitive load, represent all the high-level information in one location with a visual timeline, providing intuitive pathways to more detailed information and upcoming needed evaluation and follow-up. We took a similar approach for incorporating a patient education resource, which we designed to be readily available for the provider so that they could use it to guide real-time informed decision-making with the patient. In our field observations, we found providers using such educational materials; although they were not incorporated electronically as part of the CRC screening CDS.

Successful implementation and end-user acceptance of CDS requires high usability, low cognitive workload and integration of the CDS into workflow [12-16]. To this end, we designed a laboratory simulation experiment to understand whether our design changes to the CRC screening clinical reminder would result in improved usability, workload, and integration into workflow. We hypothesized that the redesigned CRC screening clinical reminder would be (1) perceived as easier to use, (2) perceived to have lower workload during its use, and (3) given higher ratings for workflow integration, compared to the current CRC screening clinical reminder.

## Study context

### Organizational setting and system description

The VHA includes approximately 150 medical centers and many more community based outpatient clinics in the United States. The Department of Veterans Affairs (VA) is a leader in the transition to electronic patient records and clinical decision support, including the design and implementation of computerized clinical reminders. The clinical reminders function as part of the VA's EHR, which is an integrated program with multiple software packages designed to allow providers to order medications, laboratory tests, consultations, and document actions.

The VA clinical reminder system evaluates available patient data according to a defined logic, based on the clinical topic addressed, such as CRC. If the data indicate that an intervention is potentially appropriate for the patient, the reminder is categorized as 'applicable'; if the data indicate the intervention has been provided, the clinical reminder 'satisfied' and if not provided, 'due' [17]. In the EHR, each patient's chart opens to a cover sheet where due clinical reminders are listed. Many of the reminders are nationally mandated, including the reminder for CRC screening.

**Figure 1:**
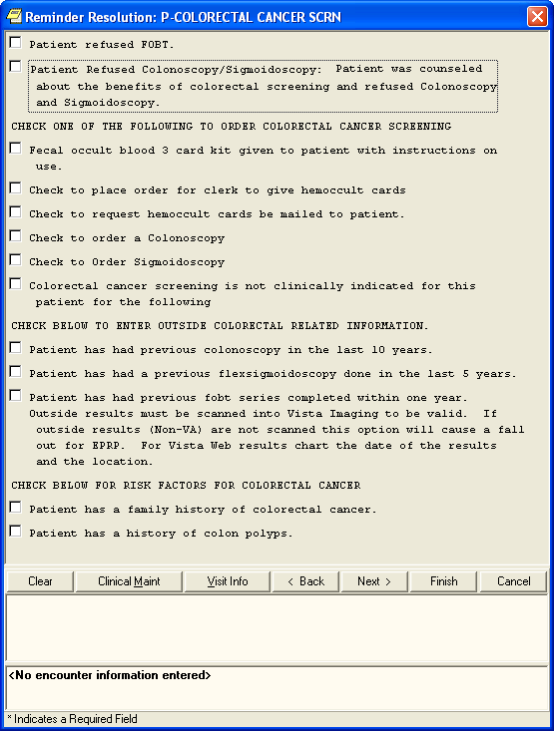
**The VHA's current computerized clinical reminder for CRC screening**.

The computerized clinical reminders in the VA's EHR are programmed to appear according to evidence-based guidelines. For example, the CRC screening reminder is triggered for patients 50 to 75 years old with no colonoscopy in the past ten years or no sigmoidoscopy in the past five years, and no record of three fecal occult blood test (FOBT) cards results reported within the past year. If the patient has a family history of colorectal cancer, the age criterion is adjusted downward to 40. If the record does include three FOBT card results in the past year, the reminder appears annually. If the record indicates that a colonoscopy was performed, the reminder will appear ten years after the colonoscopy date. Similarly, the reminder will appear five years after a sigmoidoscopy. Rank order logic is used so that the longest applicable period is chosen for the time to reappearance of this reminder. Figure 1 shows the dialog box for the current for the VHA's clinical reminder for CRC screening.

The clinicians usually view a patient's due clinical reminders just before or during a patient visit. Each outpatient exam room has a desktop computer for the provider to access the EHR and the clinical reminders. Although the clinical reminders were designed to be used during the patient visit, some providers tend to complete the reminders after the patient visit[17], which is problematic, since the provider often cannot follow the advice of the reminder after the patient has left (e.g., order a colonoscopy) and would have to defer the reminder until s/he could discuss CRC screening with the patient.

To satisfy the clinical reminders, the clinician must first create a new progress note in the EHR, then click on an icon to display a list of reminders that are due, and then click on individual reminders to invoke a dialogue box to address each one. Once the dialogue is open, the clinician satisfies a reminder by selecting the appropriate dialogue options (e.g., 'Check to order a Colonoscopy'; Figure 1). After each clinical reminder is processed, text from the completed reminder dialog box is automatically inserted in the progress note. The reminders are 'passive' in that providers have uninterrupted access to the EHR software regardless of whether they address any reminders. That is, the due reminders do not require an acknowledgment.

### System details for the prototypes

The redesigned prototype for the CRC clinic reminder was constructed using Adobe^® ^Fireworks^® ^as a low-fidelity mock-up and converted to an executable PDF file. In other words, screen captures of the current design were used as a visual base and then graphically rendered in redesigned formats. Links and buttons were made interactive to mimic the actual function of how the clinical reminders would work if fully programmed. To enable us to compare the redesign with the way the current system functions, we also "prototyped" the current system in the same fashion so that both designs were at the same simulation fidelity level. Since the prototypes used for this evaluation were mock-ups, made to mimic actual integration with the EHR, no real patient data was used to populate the CRC reminder prototypes (fictitious patient data was developed for our scenarios - see Methods section for scenario development), although the actual clinical reminder system is populated with data from the EHR.

**Figure 2:**
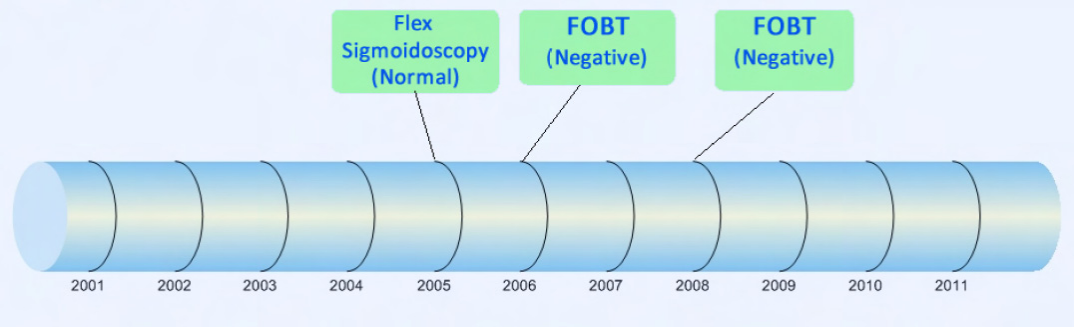
**Timeline visual that integrates previous colorectal cancer screening results**.

**Figure 3:**
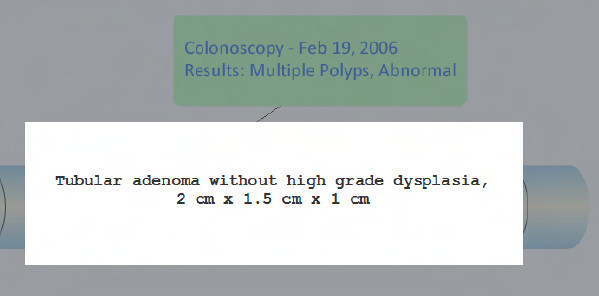
**Information for an abnormal result is displayed by clicking on the result from the timeline**.

The redesigned prototype (design B) differed from the current design (design A) in the following two ways: (1) a timeline visual was created to display a complete, integrated history of a patient's colorectal cancer screening tests and results, including FOBTs, sigmoidoscopies, and colonoscopies; and (2) a general resource (i.e., non-patient specific) was added to assist the clinician in providing patient education. Both design additions were implemented such that they could be initiated from the original reminder dialog box. Figure 2 shows an example of the timeline visual for a fictitious patient with no abnormal screening results. If a patient did have an abnormal result displayed on the timeline visual, a provider could click on the relevant test on the timeline to see specific information about that result (Figure 3). The patient education resource was added to the redesigned prototype through a link to a one-page synopsis of CRC screening [18], so that the provider could review it with the patient at the time of offering the screening.

## Methods

This study received ethical approval by the Indiana University (IU) Institutional Review Board (IRB; study # 0711-59), as well as the Indianapolis VA Medical Center Research and Development (R&D) Committee.

### Participants

We recruited 12 primary care providers (PCPs) from five outpatient clinics at the VA Medical Center study site. We considered recruiting non-VA PCPs to remove the potential bias of users having experience with the current design of the CRC clinical reminder being more likely to perform well with the current system. However, information obtained from first-time users of a system is limiting, since it would not be possible to assess whether the new design enhancements affect the performance measures after the participants move past an initial learning phase. Therefore, current VA PCPs were recruited to participate.

### Apparatus

The experiment was set-up in the Human-Computer Interaction (HCI) & Simulation Laboratory at the Indianapolis VA Medical Center. The Lab provides an environment to capture performance and usability data and assess user interaction with clinical information systems. Morae^® ^usability testing software was used to capture the direct screen image of the participant's screen in conjunction with a Web camera to record the participant's face. The experimenter was able to view the participant's screen via the Morae^® ^recording in real time from a different workstation, separated by a divider to reduce potential experimenter bias. Prototypes of the current and redesigned CRC computerized clinical reminder were displayed on the participant's computer.

### Procedure and scenarios

One of the authors (JS) facilitated the experimental sessions, including all of the procedures described in this section. Brief demonstrations of the current (design A) and redesigned (design B) prototypes were provided to orient each participant. The demonstration, including both prototypes, lasted no longer than five minutes. Each participant was introduced to designs A and B for the demonstrations in a counter-balanced fashion (i.e., design A was demonstrated for participant 1 first, design B was demonstrated for participant 2 first, and so on for all participants). Then, for the experimental protocol, each participant worked with designs A and B in the same counter-balanced order as during the demonstrations (i.e., participant 1 used design A first, participant 2 used design B first, and so on for all participants). Each participant was given brief, written instructions to resolve the CRC clinical reminder (i.e., order CRC screening and complete the rest of the reminder dialog) for two patient scenarios for each design (four total scenarios), with relevant patient information necessary to complete the reminder.

We developed a simple and complex patient scenario with the assistance of a practicing physician co-author (DH) to expose the participants to more than one type of patient case when working through the prototypes. Differences between the paired patient scenarios across designs A and B were 'surface-level' only (e.g., name, social security number) to reduce variability with user performance not related to the design of the CRC screening clinical reminder. In addition to designs A and B being presented to participants in a counter-balanced fashion, the presentation order of the simple and complex scenarios was also counterbalanced within designs A and B (i.e., participants 1 and 2 received the simple scenario first for both designs, participants 3 and 4 received the complex scenario first for both designs, and so on for all participants). An overview of the simple and complex scenarios included the following:

*• Simple: *Patient is a 60 year-old male veteran who first came to the VA about 12 months ago. He is interested in discussing colonoscopy. Prior to seeking care at the VA, he completed FOBT cards for his family physician. He thinks they were negative, but he can't remember when he had them done. In addition, he believes he had a flexible sigmoidoscopy when he turned 50. He was not sedated for the procedure.

*• Complex: *Patient is a 60 year-old male veteran who first came to the VA about 1 year ago. He wonders whether he is due for another colonoscopy. He had a colonoscopy 4 years ago. The previous colonoscopy wasn't cancer, but showed multiple polyps that the GI doctors said needed to be followed up.

The prototypes included the fictitious patients' active problem list, medications, clinical reminders, current vitals, two previous progress notes, and previous test results (including CRC screening results). Since design A did not have the new timeline visual, previous CRC screening results were available as test results, which are currently displayed in the Labs or Reports section of the EHR.

Participants completed both patient scenarios for one design (A or B) before completing the two scenarios for the other design. The computerized version of the NASA Task Load Index (TLX) [19,20] was administered to the participants after each patient scenario, a total of four times per participant. We used unweighted TLX scores as the TLX dimensional weighting procedure has been found to be of limited benefit [21,22]. Usability (Computer System Usability Questionnaire; CSUQ [23]) and workflow integration surveys (paper-based) were administered after the participant had finished both patient scenarios for a given design, a total of two times per participant. We appended the standard CSUQ usability survey with three questions specific to CRC screening. The Workflow Integration Survey was developed by our team; a description of the development of the survey and its validation is published [24]; see additional file 1: Workflow Integration Survey for a list of the 12 items that comprise the instrument. After the experimental conditions, we conducted an unstructured, open-ended debrief interview to gather additional feedback on the redesigned interface. The debrief interview was conducted to probe participants about specific issues that occurred during the scenarios to better understand comments they made while interacting with the new design features or to clarify their decision making process. Total experiment time for each participant was scheduled for a maximum of 45 minutes.

### Statistical methods

The experimental design was a within-subject 'A' (current design) vs. 'B' (redesign) comparison (the single factor was Design Type). To test the hypothesis that participants would perceive design B to be easier to use than with A, we grouped similar usability questions together. See additional file 2: Usability Survey Questions and Groupings for a list of the usability survey questions and how they were grouped. We used the non-parametric Wilcoxon Signed Ranks Test to compare the 7-point Likert-type scale (1 = strongly disagree, 7 = strongly agree) responses across A and B for the usability survey groupings. Responses to a Likert-type item are normally treated as ordinal data in which case a non-parametric test is appropriate. Responses to the NASA TLX scale (0-100) are considered interval data. Therefore, to test the hypothesis that participants would have a lower perceived workload using design B than with A, we conducted paired t-tests for each TLX item (mental demand, physical demand, temporal demand, performance, effort, and frustration level), as well as for the composite TLX score across designs A and B. Finally, to test the hypothesis that design B would receive higher ratings for workflow integration compared to design A, we first grouped the 12 questions from the Workflow Integration Survey to four subscales (navigation, functionality, ease of use, and workload). Then, as with the usability survey, we treated the data from the Workflow Integration Survey as ordinal data and used the non-parametric Wilcoxon Signed Ranks Test to compare the 5-point Likert-type scale (1 = strongly disagree, 5 = strongly agree) responses across A and B for the four subscales. All of the statistical tests were two-tailed with a 0.05 level of significance.

### Qualitative analysis

Qualitative data included the Morae^® ^video recordings of the participant sessions, an open-ended portion of the CSUQ usability survey where participants were asked to list the three most positive and negative aspects of the CRC screening clinical reminder, and the open-ended debrief interview notes. The video recordings were reviewed and all participant comments and performance-related interactions with the new design features (timeline visual and patient education resource) were compiled. Each comment or interaction with a new design feature was coded as 'positive', 'neutral', or 'negative'. The same broad coding was applied to the open-ended usability survey comments and debrief notes. Then, we integrated the findings using all of these qualitative data for common occurrences across the 12 participants (e.g., X of 12 participants expressed favorable comments for the timeline visual format of the CRC screening results).

## Results

Participant characteristics, as well as quantitative (in Tables 1 and 2) and qualitative results are presented below.

**Table 1:** Results for the Workflow Integration Survey; the 12 survey items were grouped along four subscales

Design Type	Mean	Standard Deviation	p-value (two-tailed)
*Navigation*			

Current (A)	2.5	0.9	0.011
	
Redesign (B)	3.8	0.6	

*Functionality*			

Current (A)	3.1	0.7	0.008
	
Redesign (B)	4.0	0.6	

*Ease of use*			

Current (A)	3.2	1.0	0.049
	
Redesign (B)	3.6	0.9	

*Workload*			

Current (A)	2.3	0.8	0.028
	
Redesign (B)	2.9	0.6	

**Table 2:** Results for the three items specific to CRC screening appended to the CSUQ usability survey

Usability Survey Item	Design Type	Mean	Standard Deviation	p-value (two-tailed)
'It is easy to find information about the patient's colorectal cancer screening history in this system.'	Current (A)	3.3	1.3	0.015
		
	Redesign (B)	5.2	0.9	
'It is easy to find the patient's current status with regard to colorectal cancer screening in this system.'	Current (A)	3.0	1.5	0.017
		
	Redesign (B)	5.3	0.8	

'The system provides helpful patient education materials for CRC screening.'	Current (A)	2.8	1.7	0.011
		
	Redesign (B)	5.6	0.8	

### Study population

Of the 12 PCPs who participated from the VA Medical Center study site, ten of the PCPs were physicians (9 MDs and 1 DO) and two were nurse practitioners (NPs). Participants' experience in the VHA ranged from 1 to 25 years (mean = 11.2 years; standard deviation = 7.8). The mean experience is noteworthy because the VA's EHR was implemented at the study site in 1998, about 13 years prior to the completion of this study. This indicates that average participant experience spanned the existence of the VA's EHR. Six participants had 1-10 years of experience with the VA's EHR and the other six had 11-25 years of experience. Participants with 11-25 years of experience consistency rated the usability of the current EHR higher than the participants with 1-10 years of experience on all items from the CSUQ survey. This same trend was not observed for the redesigned prototype. We suspect those with greater experience using the VA's system corresponded to higher usability due to their familiarity with the current system.

Data were not collected for two participants for the current design (A) because it took longer than expected for them to complete the scenarios with the redesigned prototype (B); we were only IRB-approved to run 45-minute sessions. Both of these participants received design B to begin the experiment based on the counter-balancing of presentation order across participants. Furthermore, we did not include the CSUQ data from the first participant because s/he misinterpreted the survey instructions to answer the questions based upon the entire EHR rather than, specifically, upon the CRC screening clinical reminder. Therefore, the results reported in Table 2 are based on 9 paired comparisons rather than 12. We clarified the CSUQ instructions after the first participant to avoid similar confusion with subsequent participants. All other data was included for the first participant; therefore, the results in Table 1 for the Workflow Integration Survey are based upon 10 paired comparisons. Finally, an experimenter error for one participant resulted in the administration of only the *Simple *scenarios for designs A and B. Therefore, results reported in Tables 1 and 2 include scores for one participant based only on using the designs with the *Simple *scenario and not the *Complex *scenario.

### Dependent measures

For the Workflow Integration Survey, design B was rated significantly higher (better) than design A for each of the four survey subscales (Table 1). Analysis revealed no significant differences in ratings for the original items in the CSUQ usability survey between designs A and B. However, PCPs rated the redesigned CRC screening reminder significantly higher (better) for the three items that were appended to the CSUQ (Table 2) by the Wilcoxon Signed Ranks Test. There were no significant differences between designs A and B for the total composite workload score or any of the individual six items for the NASA TLX.

### Qualitative results

Table 3 gives a sample of representative verbalizations related to the timeline visual of a patient's history for CRC screening. For the CRC screening timeline, six providers expressed favorable opinions for the visual format. Conversely, one provider noted that they would prefer a text format over the graphical timeline of results. An additional provider said the visual timeline should have been detailed even further, with the types of screening (colonoscopy, flexsigmoidoscopy, and FOBT) on different lines for the timeline instead of them all being reported on a single horizontal line, "...because in my mind, I have to separate those things out, because they mean different things to me in terms of what needs to be done next". The remaining four providers did not have direct feedback on the timeline visual. Five providers questioned the accuracy or reliability of the data in the timeline. That is, they questioned the ability of the timeline to reliably display the patient's screening history, especially results from outside of the VHA. Conversely, one participant said the timeline would be more reliable than patient memory (e.g., asking the patient the timing and results of their last colonoscopy). For the patient scenarios that indicated an abnormal colonoscopy result, six providers verbally expressed that the actual pathology report for the abnormal polyps was not available in the timeline. One participant determined that the pathology report was available from the timeline by clicking on the green box (see Figure 3). Two providers commented on how the timeline needed to provide additional support. For example, one provider noted, "If we have an existing recommendation by a GI [gastroenterology] doctor, it should tell me what to do in the [CRC screening clinical] reminder". Finally, two providers gave feedback that the timeline visual should be adjacent to the CRC screening clinical reminder instead of having to click another button within the reminder dialog box to see the timeline.

**Table 3:** Representative verbalizations about the timeline visual for patient history of colorectal cancer screening

*Positive*	
	"This [timeline] is really useful - they had an outside colonoscopy in 2006, there were multiple polyps removed. ... I like the design of it. I like to be able to look at a glance, especially the one that said hemocult, hemocult, hemocult [gesturing to different points in time]."

	"Yeah I'd probably use it I think it's an easier, visual way to see than the notes. And it's more reliable than patient memory. I would definitely be more likely to use something like that [timeline]. I think the timeline is easy to follow since it goes from left to right and easily highlights when it [CRC screening] was done."

	"So this is nice, I can see that he did have his colonoscopy - 2006 multiple polyps, which was abnormal. So it looks like he'll need a repeat. And that's good that this is all in one place, so I know where to go."

***Negative***	

	"You know - past information...I prefer text to anything graphical. We're just used to seeing text. I would just want to see it as a list in consecutive order."

***Further Improvements***	

	[Currently] you have all the procedures listed together on one line [in the timeline - FOBT, flex sig, colonoscopy]. I'd like to see the FOBTs separated on different line...or maybe in a different color or smaller or below the bars instead of all on the same horizontal line because in my mind I have to separate those things out because they mean different things to me in terms of what needs to be done next. So if those were separated for me in advance, it would speed my mental processing."

	"The other thing that would speed things up slightly is if the computer just calculates how many years it's been since those things have been done and throws that on there - maybe just enter the procedure that's like 2005 and it could say, 'the flex sig, 4 years ago'. And that way I won't have to do the subtraction for every item that appears there - the subtraction is already done for me."

***Reliability and Accuracy***	

	"The thing I worry about is how is that data...is it accurate? I mean how does it get in there? You know what I mean? Like one of these is an outside report and if it's an outside report, how does it get into CPRS? But assuming it was magically accurate, it would be good. I like the design of it."

	"The only thing would be you'd have to have a sense that that would be something that is very reliable so that you wouldn't have to... if it missed something somewhere for instance that would prompt me to have to go look for things on my own again. As long as it's a reliable system. Because it really just takes one episode of something like this missing something and then from then on out you're not using the tool because if it's going to miss stuff I'll just look it all up myself."

There were fewer comments regarding the patient education feature in the redesigned prototype that included the one-page synopsis of CRC screening [18]. The only recurrent finding, coded across five participants, was a preference for having a pre-printed patient education form, instead of having to click on a link to the form to print it during the patient encounter. For example, one participant noted that there were only two printers in the clinic and one works only half the time. Although not a recurrent finding across participants, one provider commented that s/he liked the patient education form, but would prefer that it not be linked to the clinical reminder for CRC screening since s/he only resolves the reminders after the patient encounter. Another provider questioned the technical quality of the patient education form, specifically, the potential resolution of the figure in the form illustrating a colonoscopy, if it were printed in black and white instead of color.

## Discussion

These findings generally supported our redesigned prototype in multiple dimensions, but were mixed. Design enhancements to the VHA's existing CRC screening clinical reminder positively impacted PCPs' workflow integration and usability ratings in terms of finding the patient's relevant data, as well as in providing helpful patient education materials. However, the redesigned prototype showed no difference in terms of perceived workload, as measured by the NASA TLX. Also, the improvements demonstrated by the usability survey were related only to the three questions specific to CRC screening design enhancements. Of these three, only the question about patient education reflected a functionality that was available in the redesigned prototype, but not in the existing reminder. Both the redesigned and current CRC clinical screening reminder may inform the patient's cancer screening history and status. The specificity of these appended items to the CSUQ regarding CRC screening was likely main reason why the results differed from the validated CSUQ survey, since the three questions added were specifically targeted to assess the issues regarding CRC screening that our design changes were meant to address.

The more general usability statements on the CSUQ about simplicity, efficiency, learnability, error recovery, overall satisfaction, and other dimensions of usability, did not produce significantly improved ratings for the resigned prototype over the current design. Adding the new design features in the redesigned prototype corresponded to additional mouse clicks and mouse movement, as recorded by the Morae software, and likely contributed to the lack of significant improvement in general usability. In terms of usability, potential advantages of the new timeline visual and patient education resource may have been off-set by the additional steps needed to access these features. These findings suggest that, while participants were supportive of the design changes to include the timeline visual and patient education resource, there is still room to improve the overall usability of the CRC screening clinical reminder and how it is integrated within the EHR.

Specific participant verbalizations provide additional insight why general usability ratings for simplicity, efficiency, and other usability constructs, were not significantly improved with the redesigned prototype. For example, two participants directly commented that the timeline visual for CRC history should be integrated at the same level as the CRC reminder (i.e., without having to click on an additional button). One participant stated: *"It would be better if you had it [the timeline] over here on the window itself [next to the reminder dialog] because otherwise you have to click back and forth and it's hard to remember. Especially which somebody with a complicated history and you got to go from one thing to another, that doesn't work. So why not put it over here, you know, same window."*

The Workflow Integration Survey received significantly higher (better) ratings for the redesigned prototype than the current design for each of the four survey subscales (navigation, functionality, ease of use, and workload). Interestingly, the NASA TLX did not show significant differences for workload. This suggests that perhaps the Workflow Integration Survey may have been more sensitive to detect differences in workload than the NASA TLX. Alternatively, 'workload' may have represented a different construct in the two instruments. The items from the Workflow Integration Survey that comprise the "workload" subscale are items 4, 8, and 12 (see additional file 1: Workflow Integration Survey). In contrast, The NASA TLX has several items that measure specific dimensions of workload: mental demand, physical demand, temporal demand, performance, effort, and frustration. There are several differences between the "workload" construct in the Workflow Integration Survey and workload as measured by the NASA TLX. The NASA TLX includes specific constructs not covered by the Workload Integration Survey subscale for workload (e.g., frustration). Also, the Workload Integration Survey does not distinguish between mental effort and physical effort. These differences suggest the instruments measure different but related constructs and may explain the difference in results between the two.

Analysis of the qualitative data revealed broad support for both the timeline visual and patient education design features. In addition, participants offered feedback about potential enhancements that may further increase acceptance and usability of these features. For example, while six of the PCP's directly expressed a positive experience with the timeline visual, five PCPs were skeptical about the reliability and accuracy of the results in the timeline. This finding represents a lack of trust in the quality or authenticity of the underlying data; potential, related solutions may be to (1) increase the quality of the underlying data and (2) transparently provide information regarding the data's source. To increase trust in the data, we envision providing additional options in the visual timeline to increase the transparency of the data. In this way, providers can investigate, for example, the specific details of a patient's colonoscopy that was performed outside of the VA. These additional details may include the contact information for the facility and physician who performed the test. The additional details may also improve the overall quality of the data.

In the case of the patient education resource, PCPs generally found the resource to be helpful, but five PCPs expressed a preference for the patient education form to be available in their clinics as a pre-printed form rather than having to access and print it from the CDS as needed. One obvious issue with our redesigned prototype was the inability of all but one PCP to recognize that the specific results of abnormal tests (i.e., colonoscopy pathology reports) were available from the timeline by clicking on a green box (see Figure 3). Provider awareness could be increased by making the box resemble a button, or provide mouse-over functionality, and to provide surface descriptors (i.e., pathology) about what data resides in the next layer of information.

These results underscore the importance of iteration in design. Ideally, more than one laboratory simulation should be conducted prior to implementation. The next logical step in the design process should be to further improve the redesigned prototype, based on the results of this study, and then to repeat the experiment to determine if further improvements to usability and perceived workload are demonstrated. In our future work, we intend to pursue these tasks: building on our current design, conducting further laboratory testing of design changes, and ultimately testing in live clinical environments.

## Limitations and challenges

It is important to note that differences observed in the usability and workflow integration measures between the current design and redesigned prototype reflect the redesigned prototype with the two designed changes as a whole; i.e., the two design changes to the redesigned prototype were not individually distinguishable in a statistical analysis. However, the qualitative analysis does provide us with specific feedback on the timeline visual and patient education resource separately. Another important limitation is that our redesigned prototype was designed largely at a conceptual level. That is, we did not attempt at this stage to test the implementation of our proposed design changes with the VHA's current Veterans Health Information System Architecture (VistA), which drives the VA's EHR. Future implementation of our proposed design changes will need to be coordinated with the future redesign of VHA's information system, which is underway.

We should also note an important challenge of the approaches we used. By relying on experienced users, we achieved certain efficiencies and likely greater input and feedback, than we would have from users with little experience. However, recruiting providers for the project was quite challenging, due to multiple competing demands. Also, VA providers are prohibited from receiving monetary compensation as an incentive for participation in VA research. Methods are needed to allow rapid usability testing and iteration, particularly from users in the field. This is an important area for both innovation and further research and development.

## Conclusion

This study demonstrates the clear value of HCI evaluation in informing the redesign of information tools such as CDS. The redesign modifications tested here were informed by previous field study[8,9] to help us first understand barriers to the use of CRC screening decision support functioning *in situ*. This type of qualitative field observation, followed by scenario-driven, comparative usability testing of experimental prototypes in a simulated setting, is complementary. Clinical software development and its uptake and integration into practice would benefit from such an approach if more widely followed. For a new CDS tool that is not yet implemented, rapid user-centered iterative design with laboratory-based simulation is critical. Adopting human factors input early and iteratively into clinical information system development can improve user performance and usability, as well as reduce cost by addressing important HCI and clinical workflow considerations pre-implementation, where the cost to redesign is much less than cost post-implementation [25]. Use of laboratory-based simulation is an excellent method to obtain these HCI data in the development of health IT.

## Competing interests

The authors declare that they have no competing interests.

## Authors' contributions

JS led the conception and design of the study, facilitated all of the experimental sessions, led the analysis and interpretations of the data, and had principal responsibility for drafting the manuscript. DH contributed to the conception and design of the study, including development of the patient scenarios; he also contributed to the interpretation of the results, as well as critically edited the manuscript. LM contributed to the conception and design of the study, the interpretation of the results, as well as critically edited the manuscript. MF contributed to the conception and design of the study, the interpretation of the results, as well as critically edited the manuscript; she also co-developed the Workflow Integration Survey for this study. CK developed the prototypes for this study, helped interpret findings, and critically edited the manuscript. NA contributed to the conception and design of the study, the interpretation of the results, as well as critically edited the manuscript; she also co-developed the Workflow Integration Survey for this study. BD is the study's principal investigator; he was responsible for the overall design and supervision of the study. He contributed to the interpretation of the results and critically edited the manuscript. All authors read and approved the final manuscript.

## Pre-publication history

The pre-publication history for this paper can be accessed here:

http://www.biomedcentral.com/1472-6947/11/74/prepub

## Supplementary Material

Additional file 1 **Workflow Integration Survey. A list of the 12 items that comprise the Workload Integration Survey**. Participants rate their responses to the items using a Likert-type scale. Click here for file

Additional file 2 **Usability Survey Questions and Groupings. A list of the usability survey questions and how they were grouped**. Click here for file

## References

[B1] Gosbee J Introduction to the human factors engineering series Jt Comm J Qual Saf 2004 30 215 219 15085787 10.1016/s1549-3741(04)30023-7

[B2] Johnson CW Why did that happen? Exploring the proliferation of barely usable software in healthcare systems Qual Saf Health Care 2006 15 Suppl 1 i76 i81 17142614 10.1136/qshc.2005.016105 PMC2464864

[B3] Karsh BT Weinger MB Abbott PA Wears RL Health information technology: fallacies and sober realities J Am Med Inform Assoc 2010 17 617 623 10.1136/jamia.2010.005637 20962121 PMC3000760

[B4] Kushniruk AW Patel VL Cognitive and usability engineering methods for the evaluation of clinical information systems J Biomed Inform 2004 37 56 76 10.1016/j.jbi.2004.01.003 15016386

[B5] Saathoff A Human factors considerations relevant to CPOE implementations J Healthc Inf Manag 2005 19 71 78 16045087

[B6] Scanlon M Computer physician order entry and the real world: we're only humans Jt Comm J Qual Saf 2004 30 342 346 15208984 10.1016/s1549-3741(04)30039-0

[B7] Zayas-Caban T Dixon BE Considerations for the design of safe and effective consumer health IT applications in the home Qual Saf Health Care 2010 19 Suppl 3 i61 i67 10.1136/qshc.2010.041897 20959321

[B8] Saleem JJ Militello LG Arbuckle N Flanagan M Haggstrom DA Linder JA Provider perceptions of colorectal cancer screening clinical decision support at three benchmark institutions AMIA Annu Symp Proc 2009 2009 558 562 20351917 PMC2815413

[B9] Doebbeling BN Saleem JJ Militello LG Flanagan M Haggstrom DA Arbuckle N Integration of computerized decision support into clinical workflow: Investigating social, technical, and contextual factors 2011 In Review

[B10] Bretthauer M Evidence for colorectal cancer screening Best Pract Res Clin Gastroenterol 2010 24 417 425 10.1016/j.bpg.2010.06.005 20833346

[B11] Gellad ZF Provenzale D Colorectal cancer: national and international perspective on the burden of disease and public health impact Gastroenterology 2010 138 2177 2190 10.1053/j.gastro.2010.01.056 20420954

[B12] Bates DW Kuperman GJ Wang S Gandhi T Kittler A Volk L Ten commandments for effective clinical decision support: making the practice of evidence-based medicine a reality J Am Med Inform Assoc 2003 10 523 530 10.1197/jamia.M1370 12925543 PMC264429

[B13] Jaspers MW Smeulers M Vermeulen H Peute LW Effects of clinical decision-support systems on practitioner performance and patient outcomes: a synthesis of high-quality systematic review findings J Am Med Inform Assoc 2011 18 327 334 10.1136/amiajnl-2011-000094 21422100 PMC3078663

[B14] Patel VL Zhang J Yoskowitz NA Green R Sayan OR Translational cognition for decision support in critical care environments: a review J Biomed Inform 2008 41 413 431 10.1016/j.jbi.2008.01.013 18343731 PMC2459228

[B15] Shiffman RN Liaw Y Brandt CA Corb GJ Computer-based guideline implementation systems: a systematic review of functionality and effectiveness J Am Med Inform Assoc 1999 6 104 114 10.1136/jamia.1999.0060104 10094063 PMC61349

[B16] Sittig DF Wright A Osheroff JA Middleton B Teich JM Ash JS Grand challenges in clinical decision support J Biomed Inform 2008 41 387 392 10.1016/j.jbi.2007.09.003 18029232 PMC2660274

[B17] Saleem JJ Patterson ES Militello L Render ML Orshansky G Asch SM Exploring barriers and facilitators to the use of computerized clinical reminders J Am Med Inform Assoc 2005 12 438 447 10.1197/jamia.M1777 15802482 PMC1174889

[B18] Torpy JM Lynm C Glass RM JAMA patient page. Colon cancer screening JAMA 2006 295 1208 10.1001/jama.295.10.1208 16522847

[B19] NASA Task Load Index (TLX) for Windows 2010 http://www.nrl.navy.mil/aic/ide/NASATLX.php Accessed on: February 10

[B20] Hart S Staveland L Development of the NASA TLX (Task Load Index) Hancock PA, Meshkati N Results from empirical and theoretical research Human Mental Workload 1988 North-Holland: Elsevier Science Publishers 139 183

[B21] Hendy KC Hamilton KM Landry LN Measuring subjective workload: When is one scale better than many? Hum Factors 1993 35 579 601

[B22] Nygren TE Psychometric properties of subjective workload measurement techniques: Implications for their use in the assessment of perceived mental workload Hum Factors 1991 33 17 33

[B23] Lewis JR IBM Computer Usability Satisfaction Questionnaires: Psychometric evaluation and instructions for use Int J Human-Comp Inter 1995 7 57 78

[B24] Flanagan M Arbuckle N Saleem JJ Militello LG Haggstrom DA Doebbeling BN Development of a workflow integration survey (WIS) for implementing computerized clinical decision support AMIA Annu Symp Proc 2011 427 34 PMC3243260 22195096

[B25] Saleem JJ Patterson ES Militello L Asch SM Doebbeling BN Render ML Using human factors methods to design a new interface for an electronic medical record AMIA Annu Symp Proc 2007 640 644 PMC2813665 18693914

