# Supplementary material for: Redesign of a computerized clinical reminder for colorectal cancer screening: a human-computer interaction evaluation
Source: BMC Med Inform Decis Mak. 2011 Nov 29;11:74. doi: 10.1186/1472-6947-11-74 (PMC3252247; doi:10.1186/1472-6947-11-74)
Supplement: Additional file 1 — Workflow Integration Survey. A list of the 12 items that comprise the Workload Integration Survey. Participants rate their responses to the items using a Likert-type scale. [file 1472-6947-11-74-S1.PDF]

Workflow Integration Survey

Please think about the work involved in using the system during the simulated patient encounters and please rate the extent to which you agree with each of the following statements. Please use the scale below where 1=strongly disagree and 5=strongly agree.

In your assessment, to what extent do you agree that:	Strongly Disagree	Disagree	Neutral	Agree	Strongly Agree	Don't know
1. Patient information is easy to find in CPRS Design 1.	1	2	3	4	5	9
2. CPRS Design 1 has all of the functions (e.g., order entry, medication list) needed to complete face-to-face patient encounters.	1	2	3	4	5	9
3. CPRS Design 1 is challenging to use.	1	2	3	4	5	9
4. Using CPRS Design 1 during face-to-face patient encounters adds effort (e.g., typing, clicks).	1	2	3	4	5	9
5. Patient information is easily accessed with CPRS Design 1.	1	2	3	4	5	9
6. CPRS Design 1 helps you perform the tasks (e.g., order entry, progress notes, record review) you need to during face-to-face patient encounters.	1	2	3	4	5	9
7. CPRS Design 1 is easy to use.	1	2	3	4	5	9
8. Using CPRS Design 1 during face-to-face patient encounters increases workload.	1	2	3	4	5	9
9. With CPRS Design 1, it is difficult to search for patient information during face-to-face encounters.	1	2	3	4	5	9
10. The same information is entered into CPRS Design 1 multiple times during face-to-face patient encounters.	1	2	3	4	5	9
11. CPRS Design 1 is frustrating to use.	1	2	3	4	5	9
12. CPRS Design 1 helps you complete face-to-face patient encounters efficiently.	1	2	3	4	5	9
