# Supplementary material for: Redesign of a computerized clinical reminder for colorectal cancer screening: a human-computer interaction evaluation
Source: BMC Med Inform Decis Mak. 2011 Nov 29;11:74. doi: 10.1186/1472-6947-11-74 (PMC3252247; doi:10.1186/1472-6947-11-74)
Supplement: Additional file 2 — Usability Survey Questions and Groupings. A list of the usability survey questions and how they were grouped. [file 1472-6947-11-74-S2.PDF]

Usability Survey: Questions and Groupings

Group 1
1. Overall, I am satisfied with how easy it is to use this system.

Group 2
19. Overall, I am satisfied with this system.

Group 3
2. It was simple to use this system.
3. I can effectively complete my work using this system.
4. I am able to complete my work quickly using this system.
5. I am able to efficiently complete my work using this system.
6. I feel comfortable using this system.
7. It was easy to learn to use this system.
8. I believe I became productive quickly using this system.
9. The system gives error messages that clearly tell me how to fix problems.
10. Whenever I make a mistake using the system, I recover easily and quickly.
11. The information provided with this system is clear.
12. It is easy to find the information I needed.
13. The information provided for the system is easy to understand.
14. The information is effective in helping me complete the tasks and scenarios.
15. The organization of the information on the systems screens is clear.
16. The interface of the system is pleasant.
17. I like using the interface of this system.
18. This system has all the functions and capabilities I expect it to have.

Appended items related specifically to colorectal cancer screening:

Group 4
20. It is easy to find information about the patient's colorectal cancer screening history in this system.

Group 5
21. It is easy to find the patient's current status with regard to colorectal cancer screening in this system.

Group 6
22. The system provides helpful patient education materials for CRC screening.

Note: Questions 1-19 comprise the Computer System Usability Questionnaire (CSUQ):
Lewis JR: IBM **Computer Usability Satisfaction Questionnaires: Psychometric evaluation and instructions for use**. *Int J Human-Comp Inter* 1995, 7:57-78.
